# The Impact of Stigma on Forensic Psychiatric Patients - a Case Report

**DOI:** 10.1192/bjo.2022.370

**Published:** 2022-06-20

**Authors:** Tharshni Umakanthan

**Affiliations:** St George's Hospital, London, United Kingdom

## Abstract

**Aims:**

The Royal College of Psychiatrists’ Fair Deal (RCPsych 2008) highlighted the far-reaching impact of stigma and discrimination on the lives of people with mental illness. The pervading nature of stigma has been acknowledged in recent national and international mental health policies (WHO). The World Health Organisation reiterates people affected by mental illness should not suffer social exclusion and marginalisation due to stigma.

**Methods:**

A sixty-year-old gentleman presented to the Emergency Department following self-inflicted stabbing to the neck resulting in pharyngeal tear and surgical repair. Previous psychiatric history included inpatient admission on Section 2 following a major depressive episode. On this admission, inpatient psychiatric review elicited three months of psychotropic medication non-adherence due to difficulties the patient had encountered in acquiring repeat medications from his GP. He had relapsed into alcohol misuse as a coping mechanism culminating in a suicide attempt at home with a knife. Upon recommencing of sertraline and risperidone during admission, he was assessed as euthymic with low risk to self. The patient had been previously abstinent from alcohol and described religion as a protective factor. Prior to discharge, the patient's GP stated he must present to the surgery in person with a form of identification. This is despite CQC guidance stipulating practices should not refuse patients registration if proof of identity cannot be produced.

**Results:**

This case illustrates the socioeconomic factors increasing likelihood of suicide including forensic history, low financial status, unstable housing and lack of social support. Substance dependence is a risk factor that can be reduced by supporting patients in accessing specialist misuse input from inpatient and community teams. The patient reported fear of stigmatisation and criminalisation which led to the avoidance of seeking treatment, deterioration in mental health and severe clinical consequences. It is imperative marginalised patients with mental illness can access quality health care and this starts with GP registration.

**Conclusion:**

Forensic mental health patients experience multiple stigmas impacting on well-being including social, institutional and media stigmatisation and high levels of internalised stigma. The Time to Change campaign focused on changing attitudes and behaviour however evaluation illustrated difficulties in tackling this issue. Healthcare professionals should be mindful to avoid stigmatising language and actions to ensure fairness in care. There is a need for the medical profession to continue striving for a tangible shift in attitudes. Advocating for those with mental illness can enhance quality of life for vulnerable patient groups.

